# Utilization of bambara groundnut (*Vigna subterranea* (L.) Verdc.) for sustainable food and nutrition security in semi-arid regions of Zimbabwe

**DOI:** 10.1371/journal.pone.0204817

**Published:** 2018-10-02

**Authors:** Juliet Mubaiwa, Vincenzo Fogliano, Cathrine Chidewe, Evert Jan Bakker, Anita R. Linnemann

**Affiliations:** 1 Department of Food Science and Technology, Chinhoyi University of Technology, Chinhoyi, Zimbabwe; 2 Food Quality and Design, Wageningen, The Netherlands; 3 Department of Biochemistry, University of Zimbabwe, Mount Pleasant, Harare, Zimbabwe; 4 Mathematical and Statistical Methods, Wageningen University, Wageningen, The Netherlands; University of Florence, ITALY

## Abstract

Bambara groundnut (*Vigna subterranea* (L.) Verdc.) is an indigenous legume crop, cultivated by subsistence farmers throughout sub-Saharan countries. Research findings indicate that the crop has great nutritional and agronomic potential, but it remains scientifically neglected. A baseline study was conducted in seven districts in semi-arid regions of rural Zimbabwe to gather knowledge on current production and utilization of bambara groundnut, assess its role in providing sustainable food and nutrition security for rural populations and determine priorities for follow-up research. Results revealed a variety of bambara groundnut processing techniques, which included boiling, soaking, roasting and milling across the surveyed districts. Reported constraints to processing and consumption included long cooking time, difficulties with milling and high firewood and water requirements. Fifty to eighty percent of respondents in all districts consumed bambara groundnut once or twice weekly from August to December. Preferred consumer attributes were taste, the satiating effect, nutritional benefits or a combination of these. Current, culturally acceptable processing techniques need improvement to support sustainable bambara groundnut processing while optimising nutrient bio-accessibility. Ultimately, community resilience to food and nutrition insecurity can be promoted by exchange of bambara groundnut processing knowledge amongst the production areas, involving the different stakeholders in the food supply chains.

## 1. Introduction

Subsistence farming and ‘cash’ cropping (i.e. crop cultivation for income generation) are the foundation of sustainable food security for rural communities in sub-Saharan Africa [1, 2]. In Zimbabwe and other sub-Saharan countries, subsistence farming depends on natural rainfall, making agricultural activities extremely sensitive to environmental changes [3, 4]. About 90% of the rural areas in Zimbabwe are situated in semi-arid agro-ecological regions (i.e. region III, IV and V) [5], which are characterised by low rainfall (≤ 800 mm), making these regions unfavourable for intensive cropping of maize, the preferred staple cereal [6].

In recent years increasing weather variability and successive droughts have resulted in decreased agricultural productivity, thus negatively affecting food security. Meanwhile, agricultural organizations and policymakers have recognized the role and untapped potential of neglected and underutilized crops (NUS) for food and nutrition security, generating income in rural areas, adapting to climate change, and mitigating climatic, agronomic and economic risks [7, 8]. Researchers recommend promoting the cultivation of indigenous, drought-tolerant crops, such as legumes, and among the proposed legumes is bambara groundnut (*Vigna subterranea* (L.) Verdc.), whose high adaptability makes it suitable for semi-arid regions where other crops fail to thrive [9]. According to Hillocks [10], the land area considered suitable for bambara groundnut cultivation is 84% for Zimbabwe, 100% for Swaziland, 98% for Uganda and 95% for Zambia.

Nutritionally, bambara groundnut represents a cheap protein-rich source that can improve the food and nutrition security status of rural households. Biochemical analysis of the carbohydrate, fat, protein and mineral content reveals that bambara groundnut produces an almost balanced diet. The nut was found to be richer in essential amino acids than groundnut [11], with a protein score of 80% as compared to 65% for groundnut, 74% for soya bean and 64% for cowpea [12]. However, like other legumes, bambara groundnut lacks sulphur-containing proteins, thus blending with a staple cereal such as maize, which contains higher levels of sulphur-containing amino acids, results in a complete food [13]. Bambara groundnut contains micronutrients such as zinc, iron, calcium and potassium. Red-seed varieties have almost twice as much iron as the cream seeds. Thus, they are especially valuable in areas where iron deficiency occurs [14].

Freshly harvested and dry bambara groundnut are consumed in many ways after processing. Freshly harvested seeds are consumed as snacks after grilling or boiling for approximately an hour [15, 16]. Dry seeds are boiled or first soaked then boiled to make a snack or porridge [15]. Dried seeds are difficult to grind due to their hard and tightly fitting seed coat. These seeds are pounded to flour, which is baked to make small flat cakes and bread [15, 17]. In Eastern Africa, bambara groundnuts are roasted, milled, and the flour is used to make a soup, a relish, and also a substitute for coffee. Additionally, a thin porridge [18, 19] and stiff porridge can be made from the flour [19]. Despite all these possible uses and the nutritional and agronomic potential, bambara groundnut remains scientifically neglected [20]. An important reason for underutilization is the hard-to-cook (HTC) phenomenon in combination with inadequate processing techniques [21]. HTC in legumes is characterized by extended cooking time (3–4 h) [19] needed to ensure adequate softening during cooking [22], and the hard seed coat makes dehulling challenging [23]. HTC in legumes is associated with structural cell modifications (e.g. autolysis of cytoplasmic organelles and lignification of middle lamella) and compositional changes (e.g. formation of insoluble pectate and interactions of proteins and phenolic compounds), which occur in the cotyledons and seed coats [21]. The HTC problem may not seem serious to people living in developed countries, who have energy resources and state-of-the-art techniques to utilize HTC legumes as well as access to a variety of protein-rich foods. However, due to the ravages of protein deficiencies, this food source is indispensable for people living in subsistence conditions who cannot afford meat [24]. According to Burchi, Fanzo [25], there are four dimensions of food security, namely food availability, food accessibility, food utilization and stability. In terms of production, bambara groundnut is managed in a traditional manner, e.g. by women who use informal seed sources and give it a low priority in land allocation [26]. Nonetheless, bambara groundnut is frequently available and accessible to rural households but underutilized because of processing bottlenecks [27, 28], causing a lack of diversity in rural diets [2]. Over the past decades, processing solutions were developed to improve the quality of local food products as well as their shelf life, but these are often not applicable for resource-limited rural communities [29]. Thus, sustainable processing approaches that fit the local social-cultural framework in rural Zimbabwe and other sub-Saharan communities are urgently needed [3, 7, 30]. Processing methods for HTC legumes used in other sub-Saharan countries, such as Ghana and Nigeria, include chemical treatments (*cooking aids*), biological treatments (*germination and fermentation*), and physical treatments (*milling*, *roasting and canning*) [21].

This paper examines the factors that affect the role of this crop in the broader context of production, processing, consumption and trade in the Zimbabwean context. The objective of the study was to take stock of the indigenous knowledge on the bambara groundnut value chains to gain insight in the way in which this protein-rich crop contributes to sustainable food and nutrition security in semi-arid regions. This research documented the current land allocation for crops and traditional processing methods for bambara groundnut in Zimbabwe. The study also assessed processing constraints and analysed the preference and consumption frequency of bambara groundnut. Specific research questions guiding the research include: (i) why do people consume bambara groundnut, and why not?, (ii) what do consumers like about this food, and what not?, (iii) what is the perception of processors on bambara groundnut processing techniques, which are the best methods and what are the problems?, (iv) how do use and processing techniques compare between districts and neighbouring countries?, and (v) does cultural background have a role on crop utilization?

## 2. Methodology

### 2.1. Study area

**Fig 1:**
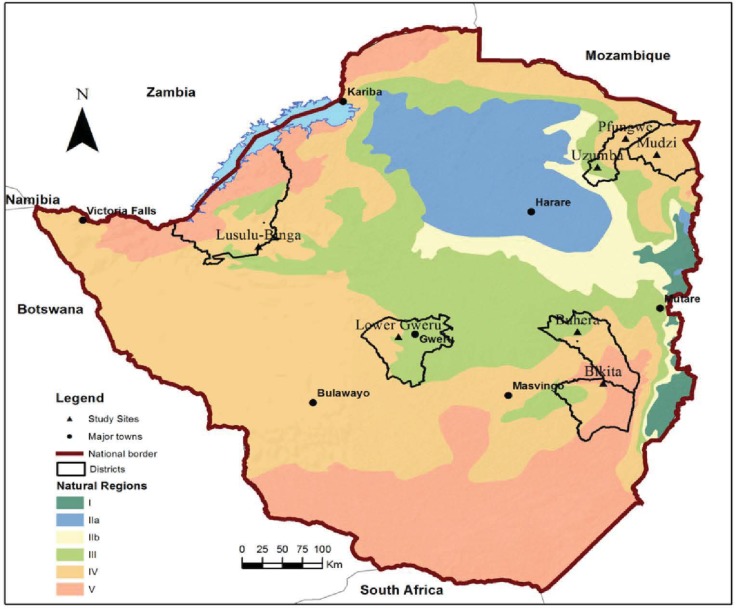
Map of Zimbabwe showing bambara groundnut surveyed districts and agro-ecological zones. Regions III (Uzumba and Binga), IV (Buhera, Mudzi, Pfungwe and Lower Gweru) and V (Bikita) receive 500–800 mm, 450–650 mm and < 450 mm, respectively.

Data were collected in rural areas in the driest parts of Zimbabwe (Fig 1), specifically in agro-ecological region III (Uzumba and Binga (Lusulu), IV (Buhera, Mudzi, Pfungwe and Lower Gweru) and V (Bikita), see Table 1. Region III receives 500–800 mm annual rainfall and is subject to seasonal droughts and severe mid-season dry spells. Region IV receives 450–650 mm annual rainfall and is characterised by frequent seasonal droughts and severe dry spells during the rainy season. Region V receives less than 450 mm annual rainfall [31]. A mixed sampling approach was applied at different stages, starting with a ‘snowball approach’ through use of network referencing [32] at Mbare farmers’ market (17° 51′ S, 31° 2′ E) in Harare (i.e. the largest informal market for agro-produce in Zimbabwe) to establish current locations of key farming areas and growers/suppliers of bambara groundnut. This was followed by judgement sampling [33] using the expert opinions of *eMKambo* (an organisation that records the movement of agro-produce in informal markets in Zimbabwe) and the Department of Research and Speciality Services (DRSS). DRSS is a government department mandated to provide research-based technologies and technical information for advisory services to support enhanced agricultural productivity. The chosen locations were amongst the major bambara groundnut growing areas in Zimbabwe and were selected for their socio-cultural diversity. The *Tonga* tribe mainly populates Binga in Matabeleland north province, whereas a mixture of *Shona* and *Ndebele* tribes populates Lower Gweru in Midlands’s province. The *Shona* tribe inhabits the rest of the districts.

**Table 1:** Demographic profile of respondents in the surveyed districts per agro-ecological region in Zimbabwe.

Region	District	Ward	N	Gender		Education		Age (years)				
				Female	Male	Educated	Not educated	19–29	30–39	40–49	50–59	60+
III	Uzumba	8	27	24	3	26	1	2	5	8	1	11
III	Binga	21	40	29	11	36	4	9	9	14	7	1
IV	Pfungwe	2	43	40	3	39	4	7	9	7	9	11
IV	Mudzi	21	30	28	2	30	0	8	8	7	3	4
IV	Buhera	14	31	31	0	29	2	2	3	7	7	12
IV	Lower Gweru	3	30	28	2	30	0	6	3	7	7	7
V	Bikita	20	30	30	0	24	6	6	2	11	2	9
3	7	7	231	210	21	214	17	40	39	61	36	55

### 2.2. Field data collection and sampling of respondents

Surveys to gather data were conducted from May to December 2014. The DRSS and the Department of Agricultural Extension Services (Agritex), both under the Ministry of Lands, Agriculture and Rural Settlement in Zimbabwe approved the study. Study areas were visited prior to the surveys for familiarization with the communities. Ward councillors, Agritex officers and village headmen were consulted before meeting household representatives to obtain verbal informed consent after explanation of research intentions and protocols. Respondents were notified of the forthcoming study and also their written informed consent was obtained before the interview. For the sampling plan, Boyd, Westfall [34] recommend a sample size of at least 5% of the total population. A sample representing 5% of the total number of households in a ward was randomly drawn from a list of households supplied by Agritex officers. A ward is defined as an administrative municipal in a district that is subdivided into villages [35]. A household was defined as all members, related or unrelated, who share the same dwelling unit. Data were collected from at least five selected villages in identified wards (ward size average 8–11 villages). With an estimated average size of 50–100 households per village, at least 5% of the households in a village was interviewed, i.e. 27–43 informants in each ward.

Data were collected through focus group discussions, formal individual interviews (see S1 Questionnaire), practical observations and demonstrations using interpreters where necessary. Table 1 shows the demographic profile of respondents for each district and agro-ecological region.

Semi-structured formal interviews were conducted with 231 respondents (of which 91% were females and 9% males) of various ages and diverse educational backgrounds. The majority of respondents (93%) had received formal education in the form of primary and secondary education. Subsistence farming, petty trade, illegal mining [36] and remittances (i.e. from relatives and food aid) were the livelihood sources of the respondents. Female respondents came from male-headed households, except the < 10% who were divorced or widowed.

The survey was split into categories to include subsistence farmers, traders (informal and formal), consumers and individual processors as shown in Table 2 [37]. Focus group discussions were conducted in Mudzi (14 participants) and Lower Gweru district (16 participants). In addition, fifty traders in Harare (from Mbare and Lusaka farmers’ markets, 17° 53'S, 30°59' E) and in Chitungwiza (from Chikwanha, 17°59' S, 31°04' E and Makoni 18° 00' S, 31° 04' E farmers’ markets) were interviewed.

**Table 2:** Type of data collected from focus group discussions and formal interviews.

Level	Data collected
Farmer level	Gender, farm size, crop prioritization (legumes and other food crops), farming inputs, harvesting and storage practices, sources of seeds, preferred seed characteristics, constraints in bambara groundnut farming, and farmer’s knowledge on the agronomical benefits of bambara groundnut cultivation.
Consumer level	General consumer information on frequency and reasons for consumption of bambara groundnut. Consumption preference (*fresh or dry seeds*), place of purchase (*e*.*g*. *informal market*, *farmers*, *and supermarket*), bambara groundnut prices relative to cowpea and groundnut. Properties considered during the buying process (*e*.*g*. *colour and size*). Preferred bambara groundnut attributes to be improved.
Processor level (individual and in groups)	Legume processing and raw material perception, processing techniques and quality of products. Sources of the bambara groundnuts, parameters considered when purchasing the seed, properties of good quality bambara groundnut ideal for processing and prices relative to cowpea and groundnut.
Trader level (formal and informal)	Places of purchase and sales of bambara groundnuts, types of purchased bambara groundnut (*fresh or dry*), consumer perception of quality seed (*e*.*g*. *colour and seed size*), criteria that determine selling price and periods of availability.

### 2.3. Data processing and analysis

Statistical analysis was done using IBM SPSS (v 23) and Microsoft Excel. Descriptive statistics (graphs / tables of arithmetic means ±SD, percentages or frequencies) are given for land allocation, bambara groundnut seed selection, production figures, legume preference, bambara groundnut based products, consumption frequency and processing constraints. Percentage land allocation per crop was calculated by taking a ratio of land (ha) allocated to that crop and the total amount of farmed land (sum of land given to this and other crops). Land allocation (ha) for bambara groundnut was tested for normality using QQ plots and variation in land allocation between regions was assessed using ANOVA at 5%level of significance. Association of the presence (yes/no) of processing constraints and firewood accessibility was tested using chi-square test of independence. All maps were constructed using QGIS Version 2.8 [38].

## 3. Results and discussion

### 3.1. Bambara groundnut production

For this study, farm size per household was categorised as small (< 2.5 ha), medium (2.5–5 ha) and large (> 5 ha) Among the different districts, most respondents (70–95%) had a medium-sized farm (2.5–5 ha), marginally different with the 0.5–2.5 ha farm size reported by Madebwe, Madebwe [39]. Diverse crops such as maize (*Zea mays*), groundnut, cowpea, bambara groundnut, as well as small grains, like sorghum (*Sorghum bicolor*), finger millet (*Eleusine coracana*) and pearl millet (*Pennisetum glaucum*), and occasionally sunflowers (*Helianthus annuus*) were grown by households as previously reported by Nhamo, Mupangwa [40] and Madebwe, Madebwe [39]. According to Makate, Wang [41], crop diversification practised by rural farmers is perceived as an ecologically feasible and cost effective way of mitigating drought and other uncertainties experienced in subsistence agriculture. As reported that subsistence agriculture is the source of livelihoods, food security and income generation in the surveyed districts [42], crop diversification brings household resilience of farming systems and nutritional diversity [41], which is a step towards increasing productivity and fighting malnutrition [43].

Table 3 shows the percentage of land allocated to each crop in each district, used as a measure of a crop’s significance in a household, either for food, income generation or both. As explained by respondents, Zimbabwe is a patriarchal society and women are not the custodians of the land [44, 45]. Hence land allocation is by men, who give preference to crops they consider indispensable [46]. However, unfavourable climatic conditions experienced in these semi-arid regions override the patriarchal factor in land allocation for maize; for example, Bikita in region V recorded the least (%) land allocated to maize.

**Table 3:** Land allocation to crops and bambara groundnut seed selection in districts.

Region	District	Average ratio of land allocation (%)*					Bambara groundnut	
		Maize	Groundnut	Cowpea	Other corps	Bambara groundnut	(ha)	% Seed selection
III	Uzumba (n = 27)	33.1 ± 9.2	25.6 ± 6.7	14.9 ± 6.5	10.8 ± 11.7	15.5 ± 5.6	0.30^ab^ ± 0.16	44.4
III	Binga (n = 40)	36.9 ± 12.8	20.5 ± 10.8	5.5 ± 7.8	9.4 ± 12.5	27.6 ± 9.9	0.53^d^ ± 0.27	82.5
IV	Pfungwe (n = 43)	34.8 ± 10.5	21.9 ± 6.3	16.1 ± 6.5	9.6 ± 10.1	17.5 ± 6.5	0.38^bc^ ± 0.21	46.5
IV	Mudzi (n = 30)	49.2 ± 6	19.9 ± 6.1	14.8 ± 4.9	0.3 ± 1.4	15.9 ± 2.9	0.21^a^ ± 0.07	93.3
IV	Buhera (n = 31)	37.3 ± 8.1	18.3 ± 8.8	9.4 ± 5.5	9.8 ± 8.3	25.3 ± 9	0.53^cd^ ± 0.21	35.5
IV	Lower Gweru (n = 29)	40.2 ± 11.2	16.2 ± 5.8	13.3 ± 6.2	10.5 ± 5.3	16.5 ± 5.2	0.21^a^ ± 0.12	60
V	Bikita (n = 30)	1.7 ± 6.7	19.8 ± 8.7	15.7 ± 6.8	31.6 ± 12.5	31.2 ± 12.4	0.60^d^ ± 0.14	16.7
	Average %	33.3 ± 2.5	20.3 ± 1.9	12.8 ± 0.9	11.7 ± 4.2	21.4 ± 3.2	0.39^bc^ ±0.233	54.1

*as percentage ratio of ha/total ha for all crops of all respondents.

Different superscript letters (a, b, c and d) in a column indicate means that are significantly different

Respondents explained that poor maize productivity led to the cultivation of small grains as their staple, which in this case accounts for 31.6% of allocated land [47]. The patriarchal factor becomes relevant in legume land allocation as reiterated by the respondents, agreeing with previous researchers, who reported bambara groundnut to be a women’s crop [48, 49]. However, Binga and Buhera districts were exceptions; here both men and women were involved in production because bambara groundnut is a cash crop to them. Results reveal that households in different districts esteem legume crops contrarily, such that Bikita, Binga and Buhera prioritize bambara groundnut, Uzumba and Pfungwe prioritize groundnut, and Mudzi and Lower Gweru allocate approximately equally areas for the three legumes. Analysis of variance (ANOVA) regarding bambara groundnut land allocation in different agro-ecological regions was not logical to perform because of unbalanced representation of regions. For this reason, analysis of variance in land allocation for crops was performed at district level, which resulted in a significant effect of district on land allocation for all crops including bambara groundnut at p < 0.05 level, e.g. bambara groundnut [F (6, 224) = 21.5, p = 0.000]. Although the sampling method was not completely randomised, it still properly represents land allocation by the populations in the regions and districts visited.

Land allocation among legumes is not a clear indicator of rainfall patterns in the districts, except for Uzumba in region III, an area considered to be good for groundnut production [40]. Land allocation for legumes seems to be governed by end use, which could be income generation, consumption or both. Those who grow bambara groundnut as a cash crop, allocate more land to its cultivation (i.e. Binga and Buhera). In contrast, cowpea is mostly grown as a dual crop for exploiting both the leaves as relish eaten with thick maize porridge and the seeds as boiled snack; this was not so for bambara groundnut [50]. The downside of bambara groundnut production as compared to cowpea was the labour intensity due to the essential ‘earthing up’ and the harvesting that requires pulling the crop out of the soil. From these findings, land allocation of maize reflects the amount of rainfall received, while land allocation for legumes reflects the significance of end use.

To produce bambara groundnut, female farmers, who are the custodians of the crop, use seeds from their previous harvest, from relatives or buy seeds in the market place [7]. Commercially produced seeds are not available [48]. Thus, sharing and using seeds from the previous crop bring convenience, community self-sustenance and the continuation of local customs that brings value to traditional systems [42]. Unfortunately, these practices sometimes result in poor germination and reduced yield as reported in some districts, agreeing with De Kock [19]. This points toward the need to expedite bambara groundnut breeding programs to improve the quality of seed sources.

Table 3 shows bambara groundnut seed selection practices in various districts, indicating that some respondents cultivate selected landraces, while others used mixed landraces, contradicting Plahar [51], who reported sole cultivation of mixed landraces in Zimbabwe. Respondents clarified the need for seed selection to be driven by some markets places. This development appears to be beneficial to farmers in terms of production as they are able to assess landrace performance in terms of drought tolerance and yield. If this is pursued over a period of time, breeders will have starting points as part of the preliminary work to develop better varieties would already have been accomplished by farmers [52].

**Fig 2:**
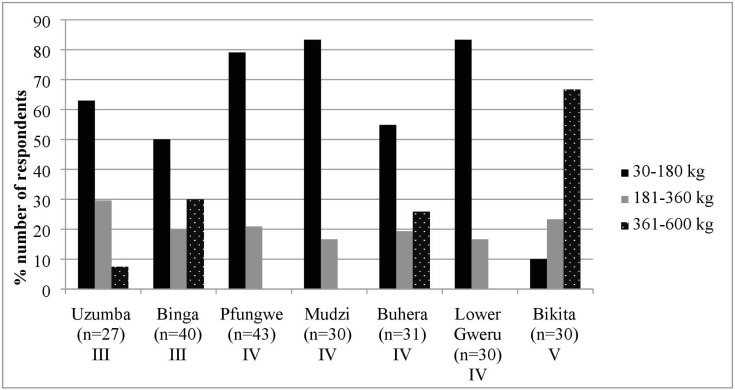
Bambara groundnut production across districts (as percentage of respondents). Shelled bambara groundnut production is categorised as low (30–180 kg), medium (181–360 kg) and high (361–600 kg) at a 60% shelling percentage.

Most respondents (63.6%) knew about the agronomical benefits of bambara groundnut farming, i.e. improved soil fertility [10], and accordingly did not use fertilizers [40]. Fortunately, bambara groundnut does well even with low input farming and poor soils [53], ensuring a definite harvest and food availability for these already financially constrained farmers [49]. The distribution of bambara groundnut production is shown in Fig 2. As expected, respondents in Bikita had a high bambara groundnut production because the district allocated more land to its cultivation. All respondents stored bambara groundnut in their shells to avoid damage by weevils.

### 3.2. Marketing bambara groundnut

Cash cropping has been suggested as a way to improve food security for smallholder farmers [2]. Local trading of bambara groundnut for income generation and exporting to neighbouring countries such as South Africa was previously reported [51, 54]According to Plahar, Annan [51], the biggest exporter of bambara groundnut in Africa is Zimbabwe with exports estimated between 1,500 and 3,000 tonnes per year. Currently, bambara groundnut is cultivated for home consumption, batter trade (i.e. exchange of goods), as income generator for a household’s sustenance or a combination of these factors, concurring with the findings of Quaye and Johnson-Kanda [49] in Ghana. Fifty to eighty-eight percent of the households sold bambara groundnut locally or at farmers’ markets and/or exported to neighbouring countries either in their capacity or through middle-persons, who link them to informal and formal markets (e.g. supermarkets). Traders in Buhera and Bikita exported to South Africa, whilst Mudzi farmers sold at Nyamapanda (a border post with Mozambique), agreeing with the reports by Plahar, Annan [51]. This shows the complexity of the bambara groundnut supply chain and the potential to improve the trading network.

The bambara groundnut trading places visited were Harare (Mbare and Lusaka farmers’ markets) and Chitungwiza (Chikwanha and Makoni farmers’ markets). The largest suppliers of bambara groundnut at these market places included farmers from Buhera, Bikita, Wedza, Uzumba, Maramba and Pfungwe districts. The traders at the market places reported all year round availability of dry bambara groundnuts, and that selling price drivers included season and supply, i.e. the seeds become more expensive towards the next planting season [55]. As previously reported by farmers, bambara groundnut sold was of mixed and individual landraces. Those who bought selected landraces typically used them for seed-fair shows or religious purposes, *viz*. especially the black-seeded landrace. As for market price, 83% of respondents reported a higher price for bambara groundnut than cowpea, whilst mixed reviews on bambara groundnut versus groundnut were given. The average selling price for bambara groundnut was 3.5 to 4 US$ per kg in local supermarkets. The recorded prices of a 20-litre bucket of dried and shelled cowpea, groundnut and bambara groundnut as of December 2015 were 10, 20 and 23 US$, respectively, at Mbare farmers’ market. The high price for bambara groundnut emphasized its importance for income generation and indicates why this crop should be given more priority.

No common criteria like a grading system for measuring the quality of bambara groundnut for trading was in place. Quality aspects considered included taste (based on previous experience), colour, size, plumpness, and absence of weevils and rot. These aspects were also reported by Mwangela, Makoka [56]. The absence of a stipulated grading system for bambara groundnut reinforces how relegated the crop is. Consequently, designing a standard grading system is yet another necessity to improve the value chain of bambara groundnut [57].

### 3.3. Processing of bambara groundnut in rural Zimbabwe

**Fig 3:**
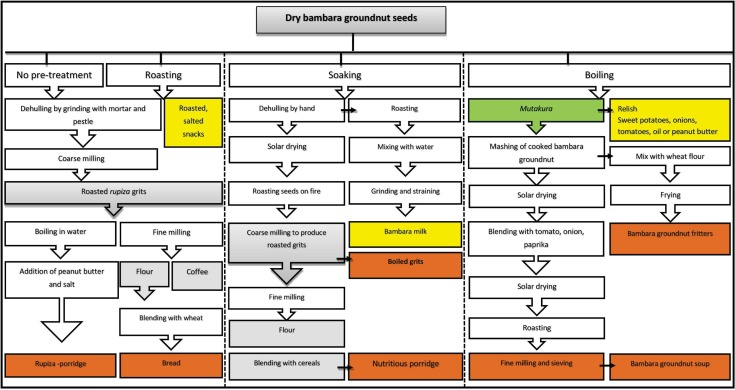
Bambara groundnut processing into various products in the semi-arid regions of Zimbabwe. The white boxes indicate processing steps and grey boxes indicate intermediate products. Green, yellow and orange are used to explain the scale of adoption and popularity of the products. Green indicates products already largely used, orange and yellow are products with minimum use. The products in orange are suggested for further improvement.

Bambara groundnut processing in rural Zimbabwe is at household level and occasionally as a collective practice by women during functions or community activities [19]. Information of processing techniques was either passed on from older family members or shared from women cooperatives. Processing methods include soaking, boiling, roasting, milling and several combinations of these methods to produce diverse products as shown in Fig 3 and Table 4, agreeing with methods from East and West African countries [17, 19].

**Table 4:** Preferred bambara groundnut-based products and their major processing practices according to agro-ecological zone and district (as percentage of respondents).

Place		Boiling					Roasting		Milling			Soaking
		Mutakura			Relish							
Region	District	snack	porridge	soup	oil	peanut	snack	coffee	flour/bread	*rupiza*	porridge	milk
III	Uzumba (n = 27)	100	0	0	0	0	11.1	40.7	7.4	33.3	0	0
III	Binga (n = 40)	100	0	0	20	7.5	0	0	0	7.5	0	0
IV	Pfungwe (n = 43)	100	7	0	48.8	34.9	32.6	0	27.9	37.2	11.6	7
IV	Mudzi (n = 30)	100	0	0	100	100	0	0	0	0	56.7	0
IV	Buhera (n = 31)	100	0	0	19.4	9.7	22.6	0	12.9	9.7	0	16.1
IV	Lower Gweru (n = 30)	100	0	73.3	93.3	100	16.7	0	0	10	0	0
V	Bikita (n = 30)	100	0	0	43.3	0	6.7	0	0	*0*	0	0

Most districts had superior processing diversity as compared to Binga and Bikita. The Binga district constitute the minority Tonga tribe who reside along the Zambezi river in the North of Zimbabwe, whilst Bikita is part of the majority Shona tribe. There are no clear cultural-processing linkages in the study, however Pfungwe, Buhera and Uzumba had older respondents who had better knowledge of processing. Simple domestic processing equipment used includes traditional grinding tools and solar tent dryers, which are designed to optimise the drying process [58].

#### 3.3.1. Boiled bambara groundnut-based products

The common method of processing bambara groundnuts in Zimbabwe is boiling in water for approximately 2–4 h to make a snack or substitute product for bread called *mutakura* [19]. Optional ingredients added during boiling include cowpea and groundnut and or maize to make an assorted *mutakura*. *Mutakura* may also be mixed with peanut butter or cooking oil and other ingredients (*viz*. tomatoes, onion, and paprika) to make a relish eaten with thick maize porridge. Additionally, *Jambalaya* is a product of *mutakura* mixed with Irish potatoes (*Solanum* sp.) or sweet potatoes (*Ipomoea batatas*).

Respondents in Pfungwe recommended porridge from mashed peanut-based *mutakura* as a complementary and weaning food for children as it is considered ‘healthy’, concurring with already published research that the legume is rich in nutrients [59, 60].

In Lower Gweru, dried bambara groundnut based soup is prepared from solar dried *mutakura* after boiling, mashing, drying and milling. The flour is mixed with fresh, grated tomatoes, onions, and spices to make dough that is further dried in a solar tent dryer to the desired moisture content. Subsequently, the dried *mutakura* mixture is roasted (until golden brown), then ground with a mortar and pestle, and finally sieved to make a soup. Lower Gweru respondents stressed the improvement of flavour and nutritional attributes after addition of the soup to relish dishes. Flavour development can be attributed to the formation of desirable flavour compounds during roasting [61].

Despite all these diverse dishes, the long cooking time involved in boiling bambara groundnut remains a major setback to its utilization as recounted by consumers (50–75%) as shown in Table 5, concurring with studies of Mwangela, Makoka [56] in Malawi and Quaye and Johnson-Kanda [49] in Ghana. Moreover, as narrated by Bressani [28] and Mubaiwa, Fogliano [21], high-energy consumption and firewood scarcity are important factors limiting bean preparation and consumption. To prove this concept, an association between the firewood accessibility constraints and processing constraints of bambara groundnut was tested. Many respondents (79.2%) with difficulties in firewood access reported constraints in processing (long cooking time), whereas 38.5% of those with easy access reported problems of processing, χ^2^(1) = 38.352, p < 0.001, N = 231. Firewood accessibility was not a limiting factor for approximately 50% of respondents in Pfungwe and Uzumba as they still had sufficient trees in their forests or had adjusted to the time it takes to boil the legume, whereas respondents in Bikita and Buhera complained of firewood shortages, indicating deforestation in the area. On the other hand, Binga and Pfungwe respondents reported shortages of potable water as a limiting factor since boiling takes a lot of water. According to the sustainable diets concept, deforestation [62, 63] and excessive water consumption are serious problems reducing the sustainability index of bambara groundnut [64]. These findings point towards the urgent need for alternative processing methods to circumvent HTC. Nevertheless, the products and recipes specified for boiled bambara groundnut offer opportunities to solve the problem of dietary diversity, which is necessary to achieve good child nutrition, considering that some dishes improve food edibility by young children [65].

**Table 5:** Constraints to bambara groundnut cooking according to agro-ecological zone and district (as percentage of respondents).

Region	District	Long cooking time	Constraint in firewood accessibility	Constraint in water accessibility
		%	%	%
III	Uzumba (n = 27)	59	55.6	0
III	Binga (n = 40)	55	0	92.5
IV	Pfungwe (n = 43)	72.1	44.2	34.9
IV	Mudzi (n = 30)	53.3	13.3	53.3
IV	Buhera (n = 31)	64.5	61.3	12.9
IV	Lower Gweru (n = 30)	53.3	80	36.7
V	Bikita (n = 30)	56.6	66.7	30

Reported remedies for circumventing HTC in the boiling processing include grading of seeds before cooking to standardise variety and or seed size because mixed seeds have dissimilar physical and water absorption properties [66, 67]. Varietal differences in processing aptitude were revealed by some respondents. Apparently, light coloured seeds have inherent thin seed coats (e.g. white) and are therefore faster to cook and mill as compared to darker landraces. These findings agree with the work of Plahar and Annan [68], who reported that in Ghana consumers who boil bambara groundnut, prefer the cream-coloured seeds (low in tannin) and choose the red-coloured seeds (high in tannin) for flour production. This finding indicates the necessity to determine the correlation between processing aptitude of varieties and nutrient indicators.

HTC can also be tackled by coarse-milling (i.e. in Pfungwe and Buhera) for size reduction prior to boiling. Consequently, the seed coat, which is a barrier to hydration, is partially removed [69] and the surface area for water absorption is increased. Additionally, HTC was addressed by pre-soaking in Bikita (33.3%), Buhera (9.7%), Mudzi and Lower Gweru (6.7%). This practice reduce the cooking time as previously reported by Annan, Plahar [60], but most respondents claimed that it also alters taste and texture. In addition, Buhera respondents (9.7%) were aware of cooking aids such as *kanwa* that is widely applied in West African countries (e.g. Ghana and Nigeria) to reduce cooking time [51, 70, 71], but were against them because of gastrointestinal problems (laxative effect) and altered sensorial attributes [72]. *Kanwa* is known as *gowa* in some parts of Zimbabwe, where it is used as a tenderizer in cooking leafy vegetables, common beans, and okra [73], but is rarely used for bambara groundnut. Since taste is the main attribute liked by bambara groundnut consumers in Zimbabwe, processing solutions that address the HTC phenomenon must not negatively impact sensorial properties.

#### 3.3.2. Alternative products

Bambara groundnuts may also be roasted to produce diverse products such as roasted snacks, *rupiza* (dehulled grits), pre-soaked *mutakura* grits and bambara groundnut milk as shown in Table 4. Roasting and soaking are pre-treatments employed prior to further processing of bambara groundnut into different products as presented in Fig 3. Soaking prior to drying, roasting and milling is employed for easy removal of the seed coats in the preparation of pre-soaked *mutakura* [9].

*Rupiza* is a product mainly of dry-roasted cowpea [74, 75] and occasionally bambara groundnuts made by coarse milling to produce grits, which are boiled and mixed with peanut butter before serving. Generally, 12% of the respondents were aware of bambara groundnut *rupiza*, with a better awareness in Uzumba and Pfungwe. Bambara groundnut *rupiza* takes 45 min to 1 h to boil and this cooking time is comparable to the boiling time of freshly harvested bambara groundnut pods [76]. Hence *rupiza* production is a solution to the problem of the long cooking time with a 75% reduction in cooking time as compared to cooking with alkaline salts [21]. However, the use of traditional milling tools is a tedious time and energy consuming process [23] due to the thick seed coat and strong attachment between the seed coat and cotyledons caused by mucilage and gums at the interface [77]. Using commercial, locally available grinding machines can facilitate this issue.

Fig 3 shows different types of flour produced in surveyed districts. The intensity of dry roasting differentiates between the production of coffee and flour; to produce coffee, dark roasting is performed. Bambara groundnut-based coffee is a substitute for tea for 80% of the respondents in Uzumba. In a study by Nti [61], an improved method of producing flour in Nigeria involves soaking seeds in water for 30 min, boiling for 25 min, and thereafter drying at 60–65°C in a hot air dryer for 10 h. Products of moist heat treatment and dehulling enhanced consumer appeal for both traditional and newly formulated bambara groundnut foods. In terms of district application, bambara groundnut and wheat flour blends are used for bread making and fritters as previously reported by De Kock [19], whilst a blend of bambara groundnut and maize flour (including other optional ingredients) is used to make a nutritious porridge. In all districts, no clear distinction for the applications of produced flour were encountered, i.e. whether flours are multipurpose or ideal for specific applications.

A minor product of roasting and boiling is the production of bambara groundnut milk previously reported by Murevanhema and Jideani [78] and Poulter and Caygill [79], whereby bambara groundnut is soaked overnight and thereafter dehulled to remove the seed coats. Dehulled bambara groundnuts are then roasted, mixed with water (ratio 1:5), crushed until the water changes in colour and the seeds have been reduced in size. Afterwards, the milk is filtered (residue removal) and boiled (optionally) to remove odours and then added to tea [78–80]. According to Poulter and Caygill [79], raw and pasteurised bambara groundnut milk contained 2.0 g protein per 100 ml.

### 3.4. Legume preference and consumption

Legume consumption preferences results, i.e., bambara groundnut, cowpea and groundnut, across districts showed that 93.3% of respondents in Lower Gweru highly ranked bambara groundnut followed by 50–60% in Bikita, Mudzi, and Buhera, whilst a minority of consumers in Pfungwe and Uzumba (25%) preferred it most. Overall, 42% of respondents prefer bambara groundnut as compared to 31% and 29% who prefer groundnut and cowpea, respectively.

**Fig 4:**
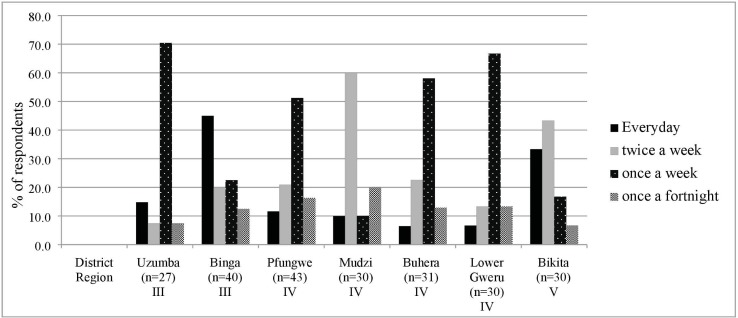
Consumption frequency of bambara groundnut in various districts (as percentage of respondents).

Consumption frequency of dry bambara groundnut in districts varied as shown in Fig 4. Respondents (50–80%) in all districts consumed bambara groundnut once to twice a week (i.e. especially from August to December). Exceptions were in Binga, where 45% of respondents reportedly consume bambara groundnut every day in summer followed by 33% in Bikita. The preferred attributes of bambara groundnut from a consumer perspective were taste, the satiating effect, its nutritional benefits or a combination of these attributes.

**Fig 5:**
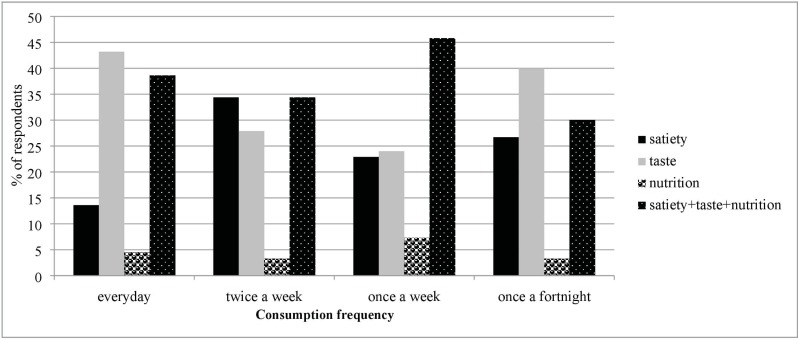
Relationship between consumption frequency of bambara groundnut and preferred attributes (as percentage of respondents).

Fig 5 shows the relationship between consumption frequency and these preferred attributes. Assessment of linkages shows that 43% of respondents who consume bambara groundnut everyday prefer its taste, whilst 38% of everyday consumers also prefer a combination of attributes (nutrition, satiety and taste). Nutrition as an independent attribute scores low, but this is not surprising as some respondents did not have any knowledge about the nutritional importance of bambara groundnut (i.e., as a source of protein). Overall, 39.4% of respondents appreciated a combination of nutrition, satiety and taste of bambara groundnut, thus any alteration in processing that distorts this is undesirable.

The relationship between consumption frequency and bambara groundnut ranking shows that respondents who highly ranked bambara groundnut were frequent consumers (i.e. 59.1% everyday consumers and 50.8% twice-a-week consumers). Nevertheless, even intermittent consumers (i.e. once a week and once a fortnight) ranked bambara groundnut fairly high, namely 45 and 48%, respectively. These frequencies show the contribution of crop to dietary diversity and food security. Unfortunately, some who consume bambara groundnuts complain of stomach pain, flatulence and general discomfort. This mostly concerns bambara groundnut and not cowpea. This could mean that bambara groundnut contains a higher amount of flatus-causing oligosaccharides than cowpea [81], implying the need to address this problem.

## 4. Conclusions and recommendations

The findings from the land allocation for crops in semi-arid districts in Zimbabwe show that farmers practise crop diversification and are aware of crops that suit their agro-ecological zones. Land allocation for legumes is governed by end use such as income generation, consumption or both. Lack of commercial seeds is a major problem in bambara groundnut farming, but a positive development of farming selected seed is identified as a step towards preliminary breeding programs to obtain drought tolerance and increased yields. The bambara groundnut value chain is characterised by the absence of stipulated grading systems, which show how relegated the crop is. Development of standard grading systems is highly recommended.

The survey showed soaking, boiling, dehulling, roasting and milling as important processing techniques for bambara groundnut in Zimbabwe, but there was no clear cultural trend on processing diversity.

The goal of legume processing is not only to solve HTC phenomena but also to retain the important sensorial and nutritional aspects of the legume-based products. The processing techniques can be evaluated from various angles, such as sustainability of the method, i.e. firewood and water consumption, processing time and aptitude, as well as the sensorial and nutritional aspects of the final product. The high-energy consumption, water and firewood scarcity are important factors limiting bambara groundnut preparation and consumption. Deforestation and excessive water consumption are serious problems reducing the sustainability index of bambara groundnut. These findings necessitate investigating the correlation between processing aptitude of processing techniques and sustainability indicators to optimise current processing methods. Appraisal of nutrient bioaccessibility and product functionality are necessary in evaluating the quality of the food products. As such, protein digestibility and micronutrient bioaccessibility studies are paramount as nutrient deficiency is still a problem that plagues sub-Saharan African communities compounded by the ravages of HIV and AIDS [6].

The limited information exchange as evidenced by variation in processing activities in various districts demonstrate the need for this exercise, i.e. among local Zimbabwe communities and in the other sub-Saharan region, for improved food security [82]. Non-governmental organizations are recommended to carry out awareness and sensitization workshops for information sharing and oversee adoption of new techniques in different areas. The government, through the ministries of agriculture, nutrition and health, is recommended to authorize policy incentives on legume farming and processing. Studies on the food technological and nutritional aspects of bambara groundnut based products are recommended. Ultimately, knowledge sharing, education, building from existing strategies and applied researches are essential interventions for improving food and nutrition security through bambara groundnut.

## Supporting information

S1 Questionnaire (DOC)

S1 Survey Data (ZIP)
