# Supplementary material for: Utilization of bambara groundnut (Vigna subterranea (L.) Verdc.) for sustainable food and nutrition security in semi-arid regions of Zimbabwe
Source: PLoS One. 2018 Oct 2;13(10):e0204817. doi: 10.1371/journal.pone.0204817 (PMC6168134; doi:10.1371/journal.pone.0204817)
Supplement: S1 Questionnaire — (DOC) [file pone.0204817.s001.doc]

Consent for Respondents

Greetings  my name is Juliet Mubaiwa and I am an enumerator for the bambara groundnut indigenous knowledge survey.  The purpose of the study is to assess the contribution of bambara groundnut farming, trading, processing and consumption towards household food security. Information on the problems encountered in the production chain from farming, processing and the end products will be collected.
As you are one of stakeholders, we would like to ask you a few questions related to this project with the intention of finding ways to improve bambara groundnut processing, consumption and subsequently food security in Zimbabwe. This information is used solely for research purposes.

Consent

I hereby confirm that I understand the contents of this document and the nature of the research project, and I consent to participating in the research project. I understand that I am at liberty to withdraw from the project at any time, should I so desire.


Signature of Respondent             		 


Table of contents
Section 1: Administration	1
Section 2: Socio-demographic characteristics	1
Section 3: Bambara groundnut farmers (To be filled by bambara groundnut farmers)	2
A.	Production patterns	2
B.	Bambara groundnut farming and storage	4
Section 4: Bambara groundnut traders	5
Bambara groundnut trading and customer preference	5
Section 5: Bambara groundnut consumers	7
A.	Bambara groundnut consumers	7
B.	Bambara groundnut processing	8
Section 6: Legume processors	9
A. (To be answered by processing companies)	9
B. (To be answered by processing cooperatives, communities and NGO etc.)	9
C. Legume processing:	10
D. Utilization of (bambara groundnut) in Zimbabwe	11
Section 7: Energy and water procurement	12

Section 1: Administration	14
Section 2: Socio-demographic characteristics	14
Section 3: Bambara groundnut farmers (To be filled by bambara groundnut farmers)	15
C.	Production patterns	15
D.	Bambara groundnut farming and storage	17
Section 4: Bambara groundnut traders	18
Bambara groundnut trading and customer preference	18
Section 5: Bambara groundnut consumers	20
C.	Bambara groundnut consumers	20
D.	Bambara groundnut processing	21
Section 6: Legume processors	23
A. (To be answered by processing companies)/ Makamba anogadzira nyimo	23
B. (To be answered by processing cooperatives, communities and NGO etc.)/ Mibatanidzwa	23
C. Legume processing:	23
D. Utilization of (bambara groundnut) in Zimbabwe	24
Section 7: Energy and water procurement	25


Section 1: Administration 
N 	Questions 	Answers 	Code 	
1.1	Name of enumerator			
1.2	Name of respondent			
1.3	Date of interview			
1.4	Time 	Start : 
Finish :		
1.5	Location 
                        	Village....................................................................
Ward.....................................................................
District...................................................................
Province.................................................................
Agro-ecological region..........................................
City.........................................................................		
1.6	Main language used by respondent	1. Shona
2. Ndebele
3. Tonga
4. English
4. Other (specify)...................................................		
1.7	Was a translater used ?	1.	Yes
2.	No		
1.8	What activities are you involved in with respect to bambara groundnut ?	1.	Farmer
2.	Trader
3.	Processor
4.	Consumer		
1.9	Identify and categorise  your main activities in 1.8  with respect to bambara groundnut.	1.............................................................................
2............................................................................
3............................................................................
4............................................................................		

Section 2: Socio-demographic characteristics
N 	Questions 	Answers 	Code 	
2.1	Gender 	1. Male
2. Female		
2.2	Location 
	Village...........................................................................................
Ward.............................................................................................
District..........................................................................................
Province........................................................................................
Agro-ecological region.................................................................
City...............................................................................................		
2.3	Ethnic group 	1. Shona
2. Ndebele
3. Tonga
4. Other (specify).........................................................................		
2.4	Marital status	1. Single
2. Married
3. Divorced
4. Widow/ widower 		
2.5	Age in years	1. ≤18
2. 19-29
3. 30-39
4. 40-49
5. 50-59
6. 60+
		
2.6	Education level	1. No education
2. Primary (Grade 7)
3. Secondary (O-Level or A-Level)
4. Vocational training
5. Tertiary (diploma or degree)
6. Other (specify).........................................................................		
2.7	Who is head of the household?	1.	Father
2.	Mother
3.	Child
4.	Grandmother
5.	Grandfather
6.	Aunt
7.	Uncle
8.	Other (specify).......................................................................		
2.8	What is your main livelihood source (source of income)?
(possible to give multiple responses in order of importance)	1.	Commercial farming 
2.	Subsistence farming
3.	Illegal mining activities
4.	Casual labour 
5.	Remittances
6.	Petty trade
7.	Other (specify).......................................................................		


Section 3: Bambara groundnut farmers (To be filled by bambara groundnut farmers)
A.	Production patterns
N 	Questions 	Answers 	Code 	
3.1	What crops do you cultivate?
(possible to give multiple responses up to three main groups in order of importance)	1.	Maize                         
2.	Groundnut
3.	Cotton                        
4.	Sorghum
5.	Cowpea                     
6.	Bambara groundnut
7.	Soya bean                
8.	Tobacco
9.	Other (Specify).....................................................		
3.2	Which one is your main crop (s) for cultivation?
(possible to give multiple responses)	1.	Maize                         
2.	Groundnut
3.	Cotton                        
4.	Sorghum
5.	Cowpea                     
6.	Bambara groundnut
7.	Soya bean                
8.	Tobacco
9.	Other (Specify)....................................................		
3.3	Who is responsible for the crops' production?	1.	Father
2.	Mother
3.	Child
4.	Grandmother
5.	Grandfather
6.	Aunt
7.	Uncle
8.	Other (specify).....................................................		
3.4	What is the source of water for your crops?
(possible to give multiple responses)	1.	Irrigation
2.	Rain-fed
3.	Other (s)………………..............................................		
3.5	What is your total land area for all farming activities in acres or hactare?
			

3.6	
Estimate the land (acres) allocated for farming of each crop?	
Maize.........................................................................
Groundnut.................................................................
Bambara groundnut..................................................
Cowpea......................................................................
Cotton........................................................................
Tobacco.....................................................................
Sorghum....................................................................
Other (specify)..........................................................		
3.7	Estimate your average yield of bambara groundnut per given area under cultivation?
(eg. how many 20 liter buckets of bambara groundnut  in shells do you harvest?	
...................................................................................		
3.8	Do you use farming inputs (fertilizer, manure) for bambara groundnut cultivation? 	1.	Yes
2.	No
If yes specify...............................................................		
3.9	Who is responsible for the farming of bambara groundnuts in the household?
(possible to give multiple responses)	1.	Father
2.	Mother
3.	Child
4.	Grandmother
5.	Grandfather
6.	Aunt
7.	Uncle
8.	Other (specify).....................................................		
3.10	What is your main source (s) of bambara groundnut seeds for farming?
(possible to give multiple responses in order of importance)	1.	Gift from relatives/friends
2.	Own retained
3.	Hybrid (agro-dealers)
4.	Government input packages/subsidies       
5.	Non-governmental organizations 
input packages
6.	Other (specify).....................................................		
3.11	Which seed colour (landrace) do you mainly cultivate? (Give traditional name of landrace)	1.	Mixed
2.	Selected 		
3.12	Estimate the period of planting to maturity of the cultivated landrace (maturation period)?	
...................................................................................		
3.13 	What is the main use (s) of harvested bambara groundnut seeds?
(possible to give multiple responses in order of importance)	1.	Consumption
2.	Selling + consumption 
		
3.14 	If answer is (2), then where do you sell/market your bambara groundnut crop produce?	1.	Local market
2.	Both local and market place		
3.15	How much of the crop do you sell (eg: 7 bags, 30 kg, 2 buckets)? (specify if its shelled or unshelled)	
..................................................................................		


B.	Bambara groundnut farming and storage

3.17 What are the agronomical (to soil) benefits of bambara groundnut farming that you know?
0. no knowledge
1. knowledgeable 
........................................................................................................................................................................................................................................................................................................................................................................................................................................................................................................................ 

3.18 Did you receive any training about the agronomical benefits of bambara groundnut farming?

1.	Yes
2.	No
If yes, then specify the source of training (Agritex, extension officers, vocational training)
................................................................................................................................................................................................................................................................................................................................................

3.19 What is the production challenges encountered in bambara groundnut farming?

1.	Cultivation (sowing, weeding)
...................................................................................................................................................................................................................................................................................................................................................................................................................................................................................................................................................................................................................................................................................................................................................................................................................................................................................................
2.	Harvesting
........................................................................................................................................................................................................................................................................................................................................................................................................................................................................................................................................................................................................................................................................................................................................................................................................................................................................

3.20	How do you store harvested bambara groundnut? (type of storage used e.g. sacks, granary)
........................................................................................................................................................................................................................................................................................................................................................................................................................................................................................................................ 
........................................................................................................................................................................

3.21	What do you use in bambara groundnut storage for preservation and why?
................................................................................................................................................................................................................................................................................................................................................
........................................................................................................................................................................

3.22     What are the challenges encountered in bambara groundnut storage?
................................................................................................................................................................................................................................................................................................................................................................................................................................................................................................................................................................................................................................................................................................


Section 4: Bambara groundnut traders
Bambara groundnut trading and customer preference
N 	Questions 	Answers 	Code 	
4.1	Place of trade	1.	Local 
2.	Both local + market places
		
4.2	Where did you obtain the bambara groundnut that you sell?
(possible to give multiple responses in order of importance)	1.	 Gift from relatives/friends
2.	Own retained
3.	Hybrid (agro-dealers)
4.	Government input packages/subsidies  
5.	Non-governmental organizations
Other (specify).....................................................		
4.3	What quality aspects do you look out for/consider when purchasing bambara groundnut for trading?

(possible to give multiple responses)	1.	Colour (red, cream, brown etc.)
Specify preference...............................................................
Reason.................................................................................

2.	Appearance (size)
Specify preference...............................................................
Reason..................................................................................

3.	Price
Specify preference...............................................................
Reason..................................................................................

4.	Quality 
i.	absence of weevils
ii.	not wilted
iii.	plumpness/ firmness
Specify preference...............................................................
Reason..................................................................................

5.	Other (specify)...............................................................		
4.4	What influences the final selling price of bambara groundnuts? 
(possible to give multiple responses)	1.	Volume of bambara groundnut produce at the market.
2.	Quality of the bambara groundnut.
3.	Prevailing market prices for the bambara groundnut (market forces).		
4.5	Do you have specific customers?	1. Yes 
2. No		
4.6	If yes, who are they?
(possible to give multiple responses)	1. Individuals             
2. Associations e.g. cooperatives, schools
Specify................................................................................... 
3. Company
  Specify................................................................................     
4. Other(specify)....................................................................
		
4.7	What are your customers' preferences when they purchase bambara groundnut seeds?
(possible to give multiple responses)	1.	Seed colour (red, cream, brown etc.)
Specify preference................................................................
Reason..................................................................................

2.	Seed size
Specify preference................................................................
Reason..................................................................................

3.	Price
Specify preference...............................................................
Reason..................................................................................

4.	Quality 
iv.	absence of weevils
v.	not wilted
vi.	plumpness/ firmness
Specify preference...............................................................
Reason..................................................................................

5.	Other (specify)...............................................................		
4.8	How do you rate the trading price of bambara groundnut relative to cowpea?
	1.	High     
2.	Low     
		
4.9	 How do you rate the trading price of bambara groundnut relative to groundnut?	1.	High     
2.	Low     
		
4.10	Which seed colour (landrace) do you mainly trade? (Give traditional name of landrace)	1.	Red
2.	Brown
3.	Cream
4.	Other Specify..................................................................
		
4.11	Do you know the quality standards related to the bambara groundnut trading?	1. Yes 
2. No

If yes, what are the standards? ..............................................................................................
		
4.12	Have you received any training on the quality standards related to the bambara groundnut trading? 	1. Yes 
2. No
If yes, specify trainer.............................................................		
4.13	Give different applications/ uses of the bambara groundnut that you know?
	
1.............................................................................................2.............................................................................................
3............................................................................................
4............................................................................................		


Section 5: Bambara groundnut consumers
A.	Bambara groundnut consumers
N 	Questions 	Answers 	Code 	
5.1	Which legumes does your household consume?
(possible to give multiple responses order of importance)	1.	Bambara groundnut
2.	Groundnut
3.	Cowpea
		

5.2	
Can you rank the pulses in terms of preference/liking?
	
1.................................................................................
2.................................................................................
3.................................................................................
4................................................................................
5.................................................................................
6.................................................................................
7.................................................................................		
5.3	Give reasons for the preference that you have outlined?	
.................................................................................		
5.4	Which type of bambara groundnut do you prefer?
(possible to give multiple responses)	1.	Fresh pods
2.	Dry seeds		
5.5	Give reasons for the choice that you have made?	
...................................................................................		
5.6	How often do you eat dry bambara groundnut? 	1.	Once a month
2.	Once a week
3.	Once in two months
4.	Everyday
5.	Other (Specify)...............................................................		
5.7	What is the source of dry bambara groundnut you consume? 
(possible to give multiple responses)	1. Own production
2. Gift from relatives or friends
3. Buying
4. Other....................................................................      		
5.8	If answer is (3), where do you buy?	1.	Local market
2.	Both local and at market place		
5.9	How do you rate the price of bambara groundnut relative to cowpea?
	1.	High     
2.	Low     
		
5.10	 How do you rate the price of bambara groundnut relative to groundnut?	1.	High     
2.	Low     

		
5.11	What parameters do you consider when buying bambara groundnut for consumption? 

(possible to give multiple responses)	Colour (red, cream, brown etc.)
Specify preference?
...................................................................................
Reason.......................................................................

Appearance (size)
Specify preference?
...................................................................................
Reason.......................................................................

Price
Specify........................................................................
Reason.......................................................................


Quality 
vii.	absence of weevils
viii.	not wilted
ix.	plumpness/ firmness
Reason.......................................................................

3.	Other 
Specify........................................................................
		


B.	 Bambara groundnut processing

5:12   How do you prepare bambara groundnut for consumption?
Fresh pods
(Ingredients, quantities, time taken, methods)
......................................................................................................................................................................................................................................................................................................................................................................................................................................................................................................................................................
......................................................................................................................................................................................................................................................................................................................................................................................................................................................................................................................................................

Dry seeds 
(Ingredients, quantities, time taken, methods)
..........................................................................................................................................................................................................................................................................................................................................................................................................................................................................................................................................................................................................................................................................................................................................................................................................................................................................................................................


5.13	Are there challenges you face in preparation of bambara groundnut?
(Preparation, resource mobilization, time taken etc.)
........................................................................................................................................................................................................................................................................................................................................................................................................................................................................................................................................................................................................................................................................................................................................................................................................................................................................................................................

5.14	 How can these challenges in bambara groundnut preparation be resolved?
........................................................................................................................................................................................................................................................................................................................................................................................................................................................................................................................................................................................................................................................................................................................................

5.15      What is your reason for consuming bambara groundnuts?
....................................................................................................................................................................................................................................................................................................................................................................
....................................................................................................................................................................................................................................................................................................................................................................

5.16 What other use(s) of bambara groundnut do you know?
..........................................................................................................................................................................................................................................................................................................................................................................................................................................................................................................................................................................................................................................................................................................................................................................................................................................................................................................................

5.17	Do you have any knowledge on nutritional value of bambara groundnuts?

1.	Yes
2.	No

If yes, then specify the importance
....................................................................................................................................................................................................................................................................................................................................................................
..................................................................................................................................................................................

5.18 What is your source of information (village health workers, clinics etc)?
..................................................................................................................................................................................
.................................................................................................................................................................................

5.19  What are the problems associated with bambara groundnut consumption?
(Side effects, discomfort)
........................................................................................................................................................................................................................................................................................................................................................................................................................................................................................................................................................................................................................................................................................................................................

Section 6: Legume processors

N 	Questions 	Answers 	Code 	
6.1	Do you process legume as ?	1.	Household
2.	Community or cooperative
3.	Company
4.	Non governmental organisation
5.	Other (Specify)...........................................................................		

A. (To be answered by processing companies)

N 	Questions 	Answers 	Code 	
6.2	What is the name of the processing company?			
6.3	How many employees does the company have? 			
6.4	When was the company established? 			

B. (To be answered by processing cooperatives, communities and NGO etc.)

N 	Questions 	Answers 	Code 	
6.5	What is the name of the processing cooperative?			
6.6	How many members does the cooperative have? 			
6.7	When was the cooperative established? 			
6.8	Why was the cooperative established?			


C. Legume processing: 
i.	Bambara groundnut 
ii.	Cowpea

N 	Questions 	Answers 	Code 	
6.9	What is the source of legume used for processing?	1. Own production             
2. Other producers/farmers  
3. Brokers /middlepersons (makoronyera)
5. GMB
6. Other (specify).................................................................................		
6.10	What are the characteristics of good raw material for processing 
(description related to the appearance, colour, size, maturity)	1.	Colour (red, cream, brown etc.)
Specify preference..............................................................
Reason...................................................................................


2.	Appearance (size)
Specify preference............................................................................. Reason...................................................................................

3.	Price
Specify...................................................................................
Reason...................................................................................

4.	Quality (absence of weevils, not wilted, plumpness/ firmness)
Specify...................................................................................
Reason...................................................................................

5.	Other 
Specify...................................................................................		
6.11	What other legumes do you process? 	1.	Cowpea
2.	Soya bean
3.	Common bean
4.	Other (specify)...................................  		


D. Utilization of (bambara groundnut) in Zimbabwe 
(This can also be cowpea)

6.12 What are the methods used to process (dry) bambara groundnut?

............................................................................................................................................................................................................................................................................................................................................................................................................................................................................................................................................................................................................................................................................................................................................................................................................................................................................................................................................................................................................................................................................................................ 

6.13 What are the problems experienced in bambara groundnut or cowpea processing?
............................................................................................................................................................................................................................................................................................................................................................................................................................................................................................................................................................................................................................................................................................................................................................................................................................................................................................................................................................................................................................................................................................................

6.14 What are the derived products?
.........................................................................................................................................................................................................................................................................................................................................................................................................................................................................................................................................................................................................................................................................................................................................................................................................................................................................................................................

6.15 What is the quality of your derived products?
..........................................................................................................................................................................................................................................................................................................................................................................................................................................................................................................................................................................................................................................................................................................................................................................................................................................................................................................................
 
6.16 Are there standards that determine quality for the derived products?
...................................................................................................................................................................................................................................................................................................................................................................
....................................................................................................................................................................................................................................................................................................................................................................

6.17 What do you want to be improved in your bambara groundnut or cowpea processing? 
................................................................................................................................................................................................................................................................................................................................................................................................................................................................................................................................................................................................................................................................................................................................................................................................................................................................................................................................................................................................................................................................................................................................................................................................................................................................................................................................................................................................................................................................................


Section 7: Energy and water procurement 
(To be answered by consumers and processors)
N 	Questions 	Answers 	Code 	
7.1	What is your source of energy for cooking and heating?
(possible to give multiple responses)	1.	Firewood
2.	Livestock and crop residues (manure)
3.	Coal
4.	Paraffin
5.	Electricity
6.	Gas
7.	Other (specify).................................................................................		
7.2	How accessible is your energy source? 	1.	Easy
2.	Fairly easy
3.	Difficult
4.	Extremely difficult		
7.3	How do you pay for energy source?
	1.	No payment
2.	Barter trade
3.	Monetary
4.	Other (specify).................................................................................		
7.4	If answer is (3), how much do you pay per given load of energy per month in US$?	
..............................................................................................................		
7.5	What is your source of water for home use?
(possible to give multiple responses)	1.	Municipal
2.	Borehole
3.	River
4.	Other (specify)..................................................................................		
7.6	How accessible is your water source?	1.   Easy
2.   Fairly easy
3.   Difficult
4.   Extremely difficult		
7.7	How do you pay for water source?
	1.	No payment
2.	Barter trade
3.	Monetary
4.	Other (specify).................................................................................		


End of questionnaire
Thank you, tatenda, siyabonga!


Mvumo ye vakapindura

Kaziwai, zita rangu ndiJuliet Mubaiwa uye ini ndiri muverengeri weongororo we chivanhu chedu maererano nekurirwa, kubikwa neudyiwa kwenyimo munyika ye Zimbabwe. Chinangwa chekudzidza ndechekuongorora zvinowanzoitwa pakurima, kushandiswa uye kugadzirwa kwenyimo mayereranone kuchengetedza kwekudya kwemhuri. Mashoko pamusoro pezvinetso zinokasanganikwa mukurima, kushandiswa uye kugadzirwa ndizvo zvichaunganidzwa.
Sezvo imie muri mumwe wevashandi, tinoda kukubvunzai mibvunzo shomanana inoenderana nebasa iri i nechinangwa chekuwana nzira dzekuvandudza kushandiswa kwevhu, kushandiswa uye nekuchengetedzwa kwezvokudya muZimbabwe. Iyi nhoroondo inoshandiswa chete nokuda kwezvinotsvakurudza. Mkasununguka kubvuma kana kuramba kuva nhengo yebasa iri

Mvumo

Ndinobvuma kuti ndanzwisisa chinangwa che basa ririrpano, uye ndinotendera kubatirana nemi.

                                   
Signature of Respondent             		 


Section 1: Administration 
N 	Questions 	Answers 	Code 	
1.1	Name of enumerator/ Zita re mubvunzi			
1.2	Name of respondent / Zita remubvunzwi			
1.3	Date of interview/ Zuva			
1.4	Time / Nguva 	Start : 
Finish :		
1.5	Location / Nzvimbo
                        	Village....................................................................
Ward.....................................................................
District...................................................................
Province.................................................................
Agro-ecological region..........................................
City.........................................................................		
1.6	Main language used by respondent/ 
Mutauro unoshandiswa nearikubvunzwa	1. Shona
2. Ndebele
3. Tonga
4. English
4. Other (specify)...................................................		
1.7	Was a translator used ?
 Pakashandiswa muturikiri here?	3.	Yes/ Hongu 
4.	No/ Kwete		
1.8	What activities are you involved in with respect to bambara groundnut ?
Munonyanyoia mabsasai maererano ne nyimo	5.	Farmer / Murimi
6.	Trader/ Mutengesi
7.	Processor/ Mugadziri or mubiki
8.	Consumer/ Mudyi		
1.9	Identify and categorise  your main activities in 1.8  with respect to bambara groundnut.
Kubva ku chitsauko 1.8 rongai nekukosha kwe mabasa amunoita	1.............................................................................
2............................................................................
3............................................................................
4............................................................................		

Section 2: Socio-demographic characteristics
N 	Questions 	Answers 	Code 	
2.1	Gender 
Mukadzi kana murume 	1. Male/ Murume
2. Female/ Mukadzi		
2.2	Ethnic group 
Muri werwudzi rwupi	1. Shona
2. Ndebele
3. Tonga
4. Other (specify).........................................................................		
2.3	Marital status
Makaroorwa here	1. Single/ Handinai kuriirwa
2. Married/ Ndakaroorwa
3. Divorced/ Ndakafirwa
4. Widow/ widower / Ndakafirwa		
2.4	Age in years
Mune makore mangani?	1. ≤18
2. 19-29
3. 30-39
4. 40-49
5. 50-59
6. 60+		
2.5	Education level
Makadzidza here uye kusvika papi?	1. No education/ Handinai kudzidza
2. Primary (Grade 7)
3. Secondary (O-Level or A-Level)
4. Vocational training
5. Tertiary (diploma or degree)
6. Other (specify).........................................................................		
2.6	Who is head of the household?
Ndiani anotonga pamusha?	9.	Father / Baba
10.	Mother/ Amai
11.	Child/ Mwana
12.	Grandmother/ Mbuya
13.	Grandfather/ Sekuru
14.	Aunt/ Tete
15.	Uncle/ Sekuru
16.	Other (specify).......................................................................		
2.8	What is your main livelihood source (source of income)?
(possible to give multiple responses in order of importance)
Munorarama nechii, taurai zvese zvinokubatsirai	8.	Commercial farming / Kurima kwepamusoro
9.	Subsistence farming/ Kurima
10.	Illegal mining activities/ Kuonga
11.	Casual labour / Maricho
12.	Remittances/ Kupiwa neveukama
13.	Petty trade/ Kutengesatengesa
14.	Other (specify).......................................................................		


Section 3: Bambara groundnut farmers (To be filled by bambara groundnut farmers)
C.	Production patterns
N 	Questions 	Answers 	Code 	
3.1	What crops do you cultivate?
(possible to give multiple responses up to three main groups in order of importance)
Munorima mbeu dzipi? (munotenderwa kutaura dzese dzamunorima)	10.	Maize / Chibage                       
11.	Groundnut/ Nzungu
12.	Cotton /Donje                       
13.	Sorghum/Zviyo
14.	Cowpea  / Nyemba                 
15.	Bambara groundnut/ Nyimo
16.	Soya bean                
17.	Tobacco/ Fodya
18.	Other (Specify).....................................................		
3.2	Which one is your main crop (s) for cultivation?
(possible to give multiple responses)
Pambeu dzenyu ndedzipi dzakakukosherai?
(munotenderwaa kupa mhinduri dzakawanda)	10.	Maize / Chibage                       
11.	Groundnut/ Nzungu
12.	Cotton /Donje                       
13.	Sorghum/Zviyo
14.	Cowpea  / Nyemba                 
15.	Bambara groundnut/ Nyimo
16.	Soya bean                
17.	Tobacco/ Fodya
18.	Other (Specify).....................................................		
3.3	Who is responsible for the crops' production?
Ndiani anotonga zvirimwa?	9.	Father / Baba
10.	Mother/ Amai
11.	Child/ Mwana
12.	Grandmother/ Mbuya
13.	Grandfather/ Sekuru
14.	Aunt/ Tete
15.	Uncle/ Sekuru
16.	Other (specify).......................................................................		
3.4	What is the source of water for your crops?
(possible to give multiple responses)
Munowana kupi mvura yekurima?
(Munotenderwa kupa mhinduri dzakawanda)	4.	Irrigation/ Kudiridzira
5.	Rain-fed/ Yemudenga
6.	Other (s)………………..............................................		
3.5	What is your total land area for all farming activities in acres or hectare?
Munda wenyu wakakura sei?
			

3.6	
Estimate the land (acres) allocated for farming of each crop?
Mungatiiudzwo kuti munorima nzvimbo yakakura sei pambeu yega yega?	
Maize/Chibage....................................................................
Groundnu/ Nzungu........................................................
Bambara groundnut/ Nyimo.........................................
Cowpea/ Nyemba..............................................................
Cotton/ Donje.....................................................................
Tobacco/ Fodya..............................................................
Sorghum/ Zviyo..............................................................
Other (specify)..........................................................		
3.7	Estimate your average yield of bambara groundnut per given area under cultivation?
(eg. how many 20 liter buckets of bambara groundnut  in shells do you harvest?
Mungatiudzawo kuti munokohwa zvakadii pambeu yega yega?	
...................................................................................		
3.8	Do you use farming inputs (fertilizer, manure) for bambara groundnut cultivation? 
Munoshandisa fertilize here mukurima?	3.	Yes/ Hongu
4.	No/ Kwete
If yes specify...............................................................		
3.9	Who is responsible for the farming of bambara groundnuts in the household?
(possible to give multiple responses)

Anotonga kurirwa kwenyimo ndiani?	9.	Father / Baba
10.	Mother/ Amai
11.	Child/ Mwana
12.	Grandmother/ Mbuya
13.	Grandfather/ Sekuru
14.	Aunt/ Tete
15.	Uncle/ Sekuru
16.	Other (specify).......................................................................		
3.10	What is your main source (s) of bambara groundnut seeds for farming?
(possible to give multiple responses in order of importance)
Mbeu yenyimo munoiwana kupi	7.	Gift from relatives/friends/ Hama neshamwari
8.	Own retained/ Dzangu ndinotengeta
9.	Hybrid (agro-dealers)/ Vatengesi
10.	Government input packages/subsidies / Kuhumumende 
11.	Non-governmental organizations 
input packages/ Ma NGO
12.	Other (specify).....................................................		
3.11	Which seed colour (landrace) do you mainly cultivate? (Give traditional name of landrace)
Munorima mbeu dzakaita sei?	3.	Mixed/ Dzakazangana
4.	Selected/ Ndinoshara		
3.12	Estimate the period of planting to maturity of the cultivated landrace (maturation period)?
Mbeu ye nyimo inoibva kwapera mwedzi mingani?	
...................................................................................		
3.13 	What is the main use (s) of harvested bambara groundnut seeds?
(possible to give multiple responses in order of importance)
Nyimo inonyanyoshandei kwamuri?	3.	Consumption/ Kudya
4.	Consumption +Selling /Kudya + kutengesa
		
3.14 	If answer is (2), then where do you sell/market your bambara groundnut crop produce?
Munotengesa kupi zvirimwa zvenyu?	3.	Local market/ Munzvimbo medu
4.	Both local and at market place/ Munzvimbo nekumusika.		
3.15	How much of the crop do you sell (eg: 7 bags, 30 kg, 2 buckets)? (specify if its shelled or unshelled)
Munotengesa zvakawanda sei?	
		


D.	Bambara groundnut farming and storage

3.17 What are the agronomical (to soil) benefits of bambara groundnut farming that you know?
Kurima nyimo kunobatsirei kuivhu?

0.	= no knowledge/ Handizivi
1.	Knowledgeable/ Ndinoziva

......................................................................................................................................................................... 

3.18 Did you receive any training about the agronomical benefits of bambara groundnut farming?
Makaziva sei kukosha kwenyimo kuivhu? 

3.	Yes / Hongu 
4.	No/ Kwete
If yes, then specify the source of training (Agritex, extension officers, vocational training) 
Tsanangurai wakabva ruzivo

................................................................................................................................................................................................................................................................................................................................................

3.19 What is the production challenges encountered in bambara groundnut farming?
Chii chinokunetsai pakurima nyimo?
4.	Cultivation (sowing, weeding) / Pakurima
........................................................................................................................................................................................................................................................................................................................................................................................................ 2. Harvesting/ Pakukohwa
.................................................................................................................................................................................................................................................................................................................................................................................. 

3.22	 How do you store harvested bambara groundnut? (type of storage used e.g. sacks, granary)
Munochengeta sei nyimo dzakakohwa?
........................................................................................................................................................................................................................................................................................................................................................................................................................................................................................................................ 
........................................................................................................................................................................

3.23	What do you use in bambara groundnut storage for preservation and why?
Munoshandisa chii kuchengetedza nyimo dzenyu?
................................................................................................................................................................................................................................................................................................................................................
........................................................................................................................................................................

3.24	What are the challenges encountered in bambara groundnut storage?
Matambudziko api amunosangana nawo pakuchengeta  nyimo dzamakohwa?
.................................................................................................................................................................................................................................................................................................................................................................................. 

Section 4: Bambara groundnut traders
Bambara groundnut trading and customer preference
N 	Questions 	Answers 	Code 	
4.1	Place of trade
Nzvimbo yekutengesera	1.	Local market/ Munzvimbo medu
2.	Both local and at market place/ Munzvimbo nekumusika..
		
4.2	What is your main source (s) of bambara groundnut seeds for farming?
(possible to give multiple responses in order of importance)
Mbeu yenyimo munoiwana kupi?	6.	Gift from relatives/friends/ Hama neshamwari
7.	Own retained/ Dzangu ndinotengeta
8.	Hybrid (agro-dealers)/ Vatengesi
9.	Government input packages/subsidies / Kuhumumende 
10.	Non-governmental organizations / Ma NGO
11.	Other (specify).....................................................		
4.3	What quality aspects do you look out for/consider when purchasing bambara groundnut for trading?
(possible to give multiple responses)

Chii chamutarisa maererano ne quality pakutengesa nyimo?	6.	Colour (red, cream, brown etc.)/ Ruvara
Specify preference...............................................................
Reason.................................................................................

7.	Appearance (size)/ Mamiriro ezvairi shanga
Specify preference...............................................................
Reason..................................................................................

8.	Price/ Mutengo
Specify preference...............................................................
Reason..................................................................................

9.	Quality / 
x.	absence of weevils/ Kusavapo kwezvipfukuto
xi.	not wilted
xii.	plumpness/ firmness/ kusimba kwadzo
Specify preference...............................................................
Reason..................................................................................

10.	Other (specify)...............................................................		
4.4	What influences the final selling price of bambara groundnuts? 
(possible to give multiple responses)
Chii chinosakisa mutengo wenyimo?	4.	Volume of bambara groundnut produce at the market./ Uwandu pamwaka
5.	Quality of the bambara groundnut/ Kuratidzira kwembeu
6.	Prevailing market prices for the bambara groundnut (market forces)/ Mutengo
		
4.5	Do you have specific customers?
Mune vanokutengerai here?	1. Yes / Hongu
2. No/ Kwete		
4.6	If yes, who are they?
(possible to give multiple responses)
Ndivanaani?	1. Individuals/ Vanhu mbune              
2. Associations e.g. cooperatives, schools/ Mubatandzwa
Specify................................................................................... 
3. Company/ Makamba
  Specify................................................................................     
4. Other(specify)....................................................................
		
4.7	What are your customers' preferences when they purchase bambara groundnut seeds?
(possible to give multiple responses)

Chii chakakoshera vatengi panyimo	1.	Colour (red, cream, brown etc.)/ Ruvara
Specify preference...............................................................
Reason.................................................................................

5.	Appearance (size)/ Mamiriro ezvairi shanga
Specify preference...............................................................
Reason..................................................................................

6.	Price/ Mutengo
Specify preference...............................................................
Reason..................................................................................

7.	Quality / 
xiii.	absence of weevils/ Kusavapo kwezvipfukuto
xiv.	not wilted
xv.	plumpness/ firmness/ kusimba kwadzo
Specify preference...............................................................
Reason..................................................................................

Other (specify)...............................................................		
4.8	How do you rate the trading price of bambara groundnut relative to cowpea?
Mutengo we nyimo mungauenzanise sei ne nyemba?
	3.	High  / Uripamsoro   
4.	Low     / Uripasi
		
4.9	 How do you rate the trading price of bambara groundnut relative to groundnut?
Mutengo we nyimo mungauenzanise sei ne nzungu?
	3.	High  / Uripamsoro   
4.	Low     / Uripasi
		
4.10	Which seed colour (landrace) do you mainly trade? (Give traditional name of landrace)
Muninyanyotengesa nyimo dzeruvara rwupi?	5.	Red/ Tsvuku
6.	Brown/ Tumbe
7.	Cream/ Chena
8.	Other Specify/ Dzimwewo..........................................
		
4.11	Do you know the quality standards related to the bambara groundnut trading?
Pane tsvakiridzo here pamaererano nenyimo?	1. Yes / Hongu
2. No/ Kwete

If yes, what are the standards? / Kana aripo mungatiudzawo here?..............................................................................................
		
4.12	Have you received any training on the quality standards related to the bambara groundnut trading? 
Pane fundiso yamakawana here maererano ne nyimo?	
1.  Yes / Hongu
2. No/ Kwete

If yes, what are the standards? / Kana aripo mungatiudzawo here?..............................................................................................
		
4.13	Give different applications/ uses of the bambara groundnut that you know?
Mungatipawo zvinoshandiswa nyimo zvamunoziva?
	
1............................................................................................
2.............................................................................................
3............................................................................................
4............................................................................................		
Section 5: Bambara groundnut consumers
C.	Bambara groundnut consumers
N 	Questions 	Answers 	Code 	
5.1	Which legumes does your household consume?
(possible to give multiple responses order of importance)
Tipei mbeu dzamunodya?	4.	Bambara groundnut/ Nyimo
5.	Groundnut/ Nzungu
6.	Cowpea/ Nyemba
		

5.2	
Can you rank the pulses in terms of preference/liking?
Munofarira dzipi?	
1.................................................................................
2.................................................................................
3.................................................................................
4................................................................................
5.................................................................................
6.................................................................................
7.................................................................................		
5.3	Give reasons for the preference that you have outlined?
Nemhakayei?	
.................................................................................		
5.4	Which type of bambara groundnut do you prefer?
(possible to give multiple responses)
Munofarira nyimo nyoro here kana dzakaoma?	3.	Fresh pods/ Nyoro
4.	Dry seeds/ Dzakaoma		
5.5	Give reasons for the choice that you have made?
Nemhakayei?	
...................................................................................		
5.6	How often do you eat dry bambara groundnut? 
Munodya nyimo kangani?	6.	Once a month/ Kamwe pamwedzi
7.	Once a week/ Kamwe pa vhiki
8.	Once in two months/ Kamwe pamwedzi miviri
9.	Everyday/ Zuva rega rega
10.	Other (Specify)...............................................................		
5.7	What is the source of dry bambara groundnut you consume? 
(possible to give multiple responses)
Nyimo dzamunodya makadziwana kupi?	1. Own production/ Tinorima
2. Gift from relatives or friends/ Takapihwa ne hama kana shamwari
3. Buying/ Takatenga
4. Other....................................................................      		
5.8	If answer is (3), where do you buy?
Makatenga kupi?	3.	Local market/ Munzvimbo medu
4.	Both local and at market place/ Munzvimbo nekumusika..		
5.9	How do you rate the trading price of bambara groundnut relative to cowpea?
Mutengo we nyimo mungauenzanise sei ne nyemba?
	1.	High  / Uripamsoro   
2.	Low     / Uripasi
		
5.10	 How do you rate the trading price of bambara groundnut relative to groundnut?
Mutengo we nyimo mungauenzanise sei ne nzungu?
	1.	High  / Uripamsoro   
2.	Low     / Uripasi
		
5.11	What parameters do you consider when buying bambara groundnut for consumption? 

(possible to give multiple responses)
Chii chamunotarisa pamunotenga nyimo?	1.	Colour (red, cream, brown etc.)/ Ruvara
Specify preference...............................................................
Reason.................................................................................

2.	Appearance (size)/ Mamiriro ezvairi shanga
Specify preference...............................................................
Reason..................................................................................

3.	Price/ Mutengo
Specify preference...............................................................
Reason..................................................................................

4.	Quality / 
xvi.	absence of weevils/ Kusavapo kwezvipfukuto
xvii.	not wilted
xviii.	plumpness/ firmness/ kusimba kwadzo
Specify preference...............................................................
Reason..................................................................................

Other (specify)...............................................................		


D.	 Bambara groundnut processing

5:12   How do you prepare bambara groundnut for consumption?
Fresh pods/ Nyoro
(Ingredients, quantities, time taken, methods)
......................................................................................................................................................................................................................................................................................................................................................................................................................................................................................................................................................
......................................................................................................................................................................................................................................................................................................................................................................................................................................................................................................................................................

Dry seeds / Dzakaoma
(Ingredients, quantities, time taken, methods)
..........................................................................................................................................................................................................................................................................................................................................................................................................................................................................................................................................................................................................................................................................................................................................................................................................................................................................................................................


5.15	Are there challenges you face in preparation of bambara groundnut?
(Preparation, resource mobilization, time taken etc.)
Munosangana nematambudziko api pakubika nyimo?
........................................................................................................................................................................................................................................................................................................................................................................................................................................................................................................................................................................................................................................................................................................................................................................................................................................................................................................................

5.16	 How can these challenges in bambara groundnut preparation be resolved?
Zvingagadziriswe sei?
........................................................................................................................................................................................................................................................................................................................................................................................................................................................................................................................................................................................................................................................................................................................................

5.17	What is your reason for consuming bambara groundnuts?
Nemhakayei muchifarira kudya nyimo?
....................................................................................................................................................................................................................................................................................................................................................................
....................................................................................................................................................................................................................................................................................................................................................................

5.18	What other use(s) of bambara groundnut do you know?
Nyimo dzinoshanda chii chimwe chamunoziva?
..........................................................................................................................................................................................................................................................................................................................................................................................................................................................................................................................................................................................................................................................................................................................................................................................................................................................................................................................


5.18	Do you have any knowledge on nutritional value of bambara groundnuts?
Ko pahutano pane zvamoziva here?

3.	Yes/ Hongu
4.	No/ Kwete

If yes, then specify the importance
....................................................................................................................................................................................................................................................................................................................................................................
..................................................................................................................................................................................

5.19	What is your source of information (village health workers, clinics etc)?
Makaziva sei?
..................................................................................................................................................................................
.................................................................................................................................................................................

5.19  What are the problems associated with bambara groundnut consumption?
(Side effects, discomfort)
Chii chinokunetsai kana madya nyimo?
........................................................................................................................................................................................................................................................................................................................................................................................................................................................................................................................................................................................................................................................................................................................................


Section 6: Legume processors

N 	Questions 	Answers 	Code 	
6.1	Do you process legume as ?
Munogadzira nyimo se?	6.	Household/ Mhuri yedu
7.	Community or cooperative/ Mubatanidzwa
8.	Company/ Kamba
9.	Non-governmental organisation
10.	Other (Specify)...........................................................................		

A. (To be answered by processing companies)/ Makamba anogadzira nyimo

N 	Questions 	Answers 	Code 	
6.2	What is the name of the processing company?
Bato renyu rinonzi ani?			
6.3	How many employees does the company have? 
Mune vashandi vangani?			
6.4	When was the company established? 
Makatanga riini kamba?			

B. (To be answered by processing cooperatives, communities and NGO etc.)/ Mibatanidzwa

N 	Questions 	Answers 	Code 	
6.5	What is the name of the processing cooperative?
Bato renyu rinonzi chii?			
6.6	How many members does the cooperative have? 
Muri vangani?			
6.7	When was the cooperative established? 			
6.8	Why was the cooperative established?
Makariumba riini?			

C. Legume processing: 
iii.	Bambara groundnut / nyimo
iv.	Cowpea/ nyemba

N 	Questions 	Answers 	Code 	
6.9	What is the source of legume used for processing?
Nyimo munodziwanepi?	1. Own production/ Tinorima
2. Gift from relatives or friends/ Takapihwa ne hama kana shamwari
3. Buying/ Takatenga
4. Other....................................................................      		
6.10	What are the characteristics of good raw material for processing 
(description related to the appearance, colour, size, maturity)	1.	Colour (red, cream, brown etc.)/ Ruvara
Specify preference...............................................................
Reason.................................................................................

2.	Appearance (size)/ Mamiriro ezvairi shanga
Specify preference...............................................................
Reason..................................................................................
3.	Price/ Mutengo
Specify preference...............................................................
Reason..................................................................................

4.	Quality / 
i.	absence of weevils/ Kusavapo kwezvipfukuto
ii.	not wilted
iii.	plumpness/ firmness/ kusimba kwadzo
Specify preference...............................................................
Reason..................................................................................

5.	Other (specify)...............................................................		


D. Utilization of (bambara groundnut) in Zimbabwe 


6.12 What are the methods used to process (dry) bambara groundnut?
Munogadzira sei nyimo?

............................................................................................................................................................................................................................................................................................................................................................................................................................................................................................................................................................................................................ 
6.13 What are the problems experienced in bambara groundnut or cowpea processing?
Munosangana nematambudziko api?
................................................................................................................................................................................................................................................................................................................................................................................................................................................................................................................................................................................................................................................................................................................................................................................................................

6.14 What are the derived products?
Munogadzira chii?
............................................................................................................................................................................................................................................................................................................................................................................................................................................................................................................................................................................................................ 
6.15 What is the quality of your derived products?
Zvigadzirwa zvenyu zvakamira sei?
............................................................................................................................................................................................................................................................................................................................................................................................................................................................................................................................................................................................................ 

6.16 Are there standards that determine quality for the derived products?
Chii chinotaridxa kusvika kwe zvagadzirwa nenyimo?
...................................................................................................................................................................................................................................................................................................................................................................

11.17	What do you want to be improved in your bambara groundnut processing? 
Chii chamungade chigadziriswe pakugadzirwa kwenyimo? ...................................................................................................................................................................................................................................................................................................................................................................

Section 7: Energy and water procurement 
(To be answered by consumers and processors)
N 	Questions 	Answers 	Code 	
7.1	What is your source of energy for cooking and heating?
(possible to give multiple responses)
Munobika nyimo nei?	8.	Firewood/ Huni
9.	Livestock and crop residues (manure)/ Manyowa 
10.	Coal
11.	Paraffin
12.	Electricity/ Magetsi 
13.	Gas
14.	Other (specify).................................................................................		
7.2	How accessible is your energy source?
Zviri nyore here kuwana huni? 	5.	Easy/ Nyore 
6.	Difficult/ Zvakaoma
		
7.3	How do you pay for energy source?
Munobhadhara here kuti muwane huni?	5.	No payment/ Kwete
6.	Yes/ Hongu		
7.4	If answer is (3), how much do you pay per given load of energy per month in US$?
Mungatiudzwo mutengo wacho?	
..............................................................................................................		
7.5	What is your source of water for home use?
(possible to give multiple responses)
Mvura yekubikisa munoiwanepi?	5.	Municipal/ Kanzuru
6.	Borehole/ Chibhorani
7.	River/ Rwizi
8.	Other (specify)..................................................................................		
7.6	How accessible is your water source?
Zviri nyore here kuwana mvura?	1.	Easy/ Nyore 
2.	Difficult/ Zvakaoma
		
7.7	How do you pay for water source?
Munobhadhara mvura here?
	5.	No payment/ Kwete
6.	Yes/ Hongu		


End of questionnaire
Thank you, tatenda, siyabonga!
